# Multiple Consecutive Infections Might Explain the Lack of Protection by BCG

**DOI:** 10.1371/journal.pone.0094736

**Published:** 2014-04-16

**Authors:** Pere-Joan Cardona, Cristina Vilaplana

**Affiliations:** 1 Unitat de Tuberculosi Experimental, Fundació Institut d'Investigació en Ciències de la Salut Germans Trias i Pujol, Badalona, Catalonia, Spain; 2 Universitat Autònoma de Barcelona (UAB), Bellaterra, Catalonia, Spain; 3 Centro de Investigaciones Biomédicas En Red Enfermedades Respiratorias (CIBERES), Madrid, Spain; University of Padova, Medical School, Italy

## Abstract

Although contacts between tuberculosis patients may result in multiple consecutive infections (MCI), no experimental animal models consider this fact when used in basic studies. Moreover, the current TB vaccine (BCG) has demonstrated a limited protection in humans. In this study we evaluate the effect of tuberculosis MCI by way of a simple mathematical analysis using data from the low dose aerosol murine experimental model. The results show that a higher number of, or shorter intervals between, multiple consecutive infections reduce the protective effect of BCG. This is due to both the increase in bacillary load at the stationary level of the infection, and the protective immune response induced by the infection itself. This factor must therefore be taken into account when designing new prophylactic strategies as candidate vaccines for the replacement of BCG.

## Introduction

Despite being an ancient disease and the enormous efforts devoted to its study, there are still many unknowns surrounding tuberculosis (TB). One of the main difficulties in controlling active disease (TB) is that once *Mycobacterium tuberculosis* (Mtb) infection has occurred, only 10% of infected subjects will go on to develop TB over a very variable length of time [1], although the highest risk of this happening is during the first two years postinfection. This is the reason for the “contact tracing” activity classically undertaken once a case of TB has been identified [2]: the people with whom the infected person has come into close contact are investigated, usually by performing a Tuberculin Skin Test (TST) or T-cell Interferon Gamma Release Assay (TIGRA) [3], to see whether they are also infected. Although very useful, these techniques are not able to discern how many times a contact case is infected. In the “contact tracing” strategy, three risk circles are usually drawn according to the number of hours of contact with the TB patient—the higher the number of contact hours, the higher the risk of becoming infected [2]. However, does this mean that infection is a single event and the number of hours simply reflects a higher probability of this single event happening? Currently it cannot be demonstrated that, once infected, the infected subject avoids another infection. Indeed, it is logical to suppose that this situation includes a population in an asymmetric distribution, with some people suffering “multiple consecutive infections” (MCI) of greater or lesser intensity. This is very relevant for two reasons. First of all because this will have an effect on the global infective dose of the subject; and secondly because Mtb infection generates a specific immune response as good as that induced by BCG [4].

One of the main objectives in the fight against TB over the past 20 years has been to develop a prophylactic vaccine that gives better protection than the Bacillus Calmette Guerin (BCG), which is widely used and administered to neonates despite having only a limited effect [5]. Unfortunately, the most developed candidate, known as MVA-Ag85, was recently shown to be unable to induce further protection [6], thereby suggesting the need to rethink prophylactic TB vaccine strategies [7], [8].

In this regard, and in light of the usual preclinical assays for the development of such vaccines [8], it is interesting to note that, until very recently [7], no attention has ever been paid to testing the influence of MCI, even for the gold standard BCG. This means that in preclinical assays, and despite the large variety of experimental models used, all vaccines are tested against only a single infection. In particular, the low dose (about 100 bacilli) aerosol infection in mice, which achieves an exponential bacillary growth in lungs that reaches a stationary level on day 20 post-challenge, is the most widely used [9]. In this context, BCG vaccination provides a protection based on a 1-log reduction in pulmonary bacillary load from day 15 onwards due to an earlier accumulation of Mtb-specific Th1 lymphocytes in the lesions [10]. This is important because it is thought that lower stationary level leads to lower risk of developing active TB, although this concept is currently being challenged [7].

In this paper we evaluate the protection conferred by BCG vaccination in naïve mice against a process of “multiple consecutive infections”. We believe that this process better resembles what happens in the case of close contact with an active TB case. Our findings highlight the need to address this issue in preclinical experimental modelling when testing new TB vaccine candidates.

## Material And Methods

### The growth model

The following exponential equation was used in our study:
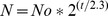
where *No* is the initial dose (100 CFUs), and *t* is the time in days.

Data were analysed using a spreadsheet (Microsoft Excel 2010).

### Parameters for the infection evolution

1.-The total bacillary load in the lung. This indirectly reflects the level of bacillary concentration in the lymph nodes. It is assumed that the immune response is triggered once a certain concentration threshold is exceeded [11].

2.- The local bacillary load at each infection site. It is assumed that once a certain threshold concentration is reached at a site, there is sufficient capacity to attract the specific lymphocytes that will be needed to control the bacillary load [11].

**Figure 1:**
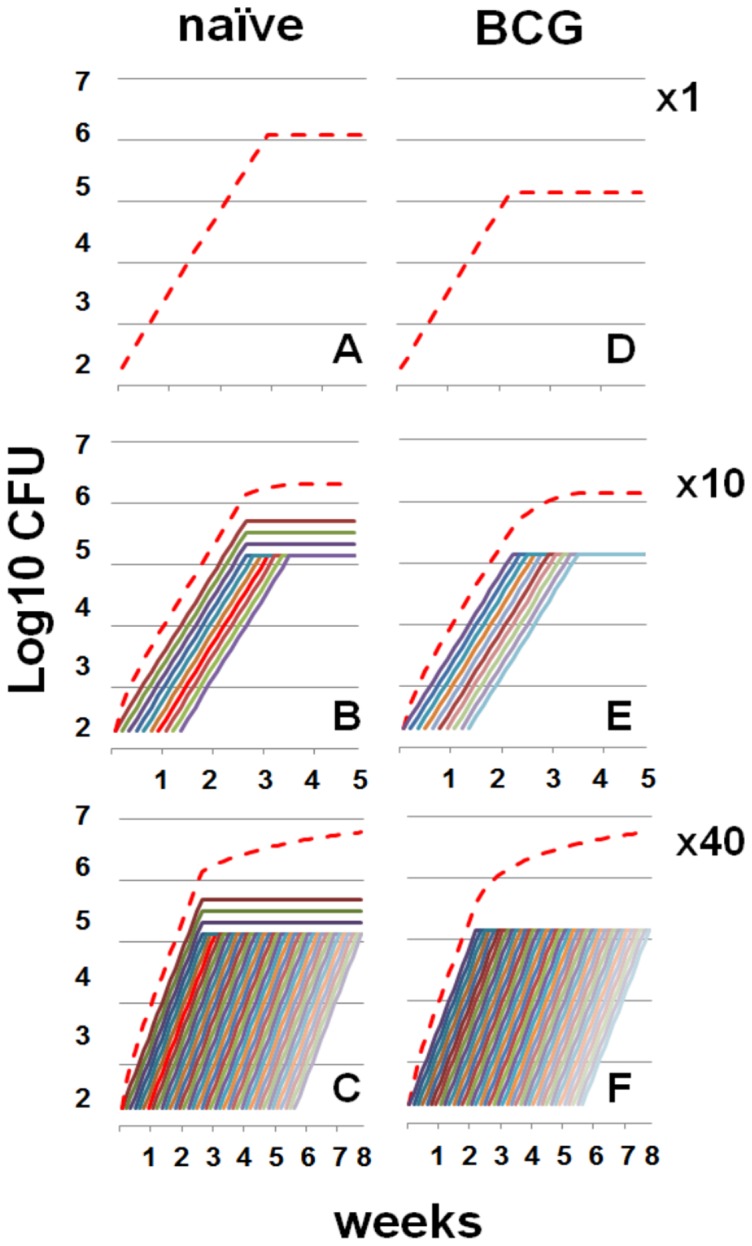
Evolution of the bacillary load in the model of single or multiple consecutive infections (MCI). Progression of the bacillary concentration in both naïve (A to C) and BCG-vaccinated mice (D to F) after single infection (A and B) or MCI with 10 (x10) or 40 (x40) infections. The red line represents the sum of the bacillary load at all individual infection sites.

3.- Stationary level. This is reached when the bacillary load at an infection site stabilizes. It is the final result of the previous parameters and requires a certain bacillary load in the lymph nodes, which is lower in vaccinated mice, to trigger the immune response; and a certain bacillary load at the infection site to attract the specific lymphocytes. In the case of a single infection this parameter reaches about 6 log (1,195,374 bacilli precisely) at day 20 post-infection in naïve animals but five days earlier and with a lower bacillary concentration (135,952 bacilli, about 5 logs) in BCG-vaccinated animals (Figure 1). This resembles data obtained in experimental in vivo modelling [10].

### Model of multiple consecutive infections

The time intervals considered were:

- every 24 hours for 1, 3, 5, 10, 15, 20, 40 or 80 days
- a total of 10 consecutive infections, one every 1.5, 3, 6, 12 or 24 hours

The total bacillary load at each time interval was calculated as the sum of the bacillary load achieved as a result of replication of previous infections plus the new infections.

## Results

### A higher number of MCI results in a lower protective effect of BCG

**Figure 2:**
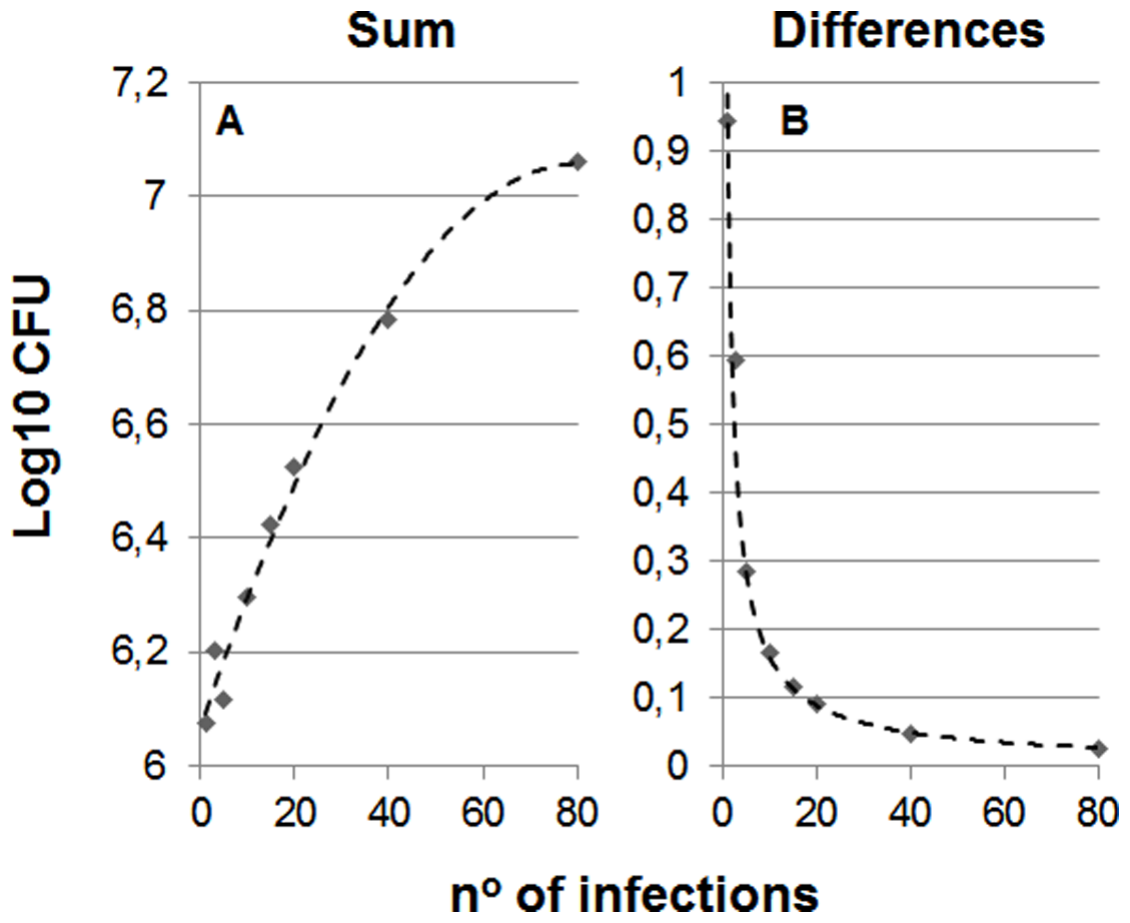
Influence of the number of multiple consecutive infections (MCI) to the stationary level. Picture A shows the evolution of the total bacillary load in the lung (sum) after different number of MCI in naïve mice. The data have been adjusted to a polynomic formula: 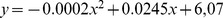 (R^2^ = 0.9865). In Picture B which shows differences between naïve and vaccinated mice, the formula has been adjusted to an exponential one: 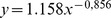 (R^2^ = 0.9883).

It can be seen from Figures 1 and 2, which show the effect of MCI, that a higher number of infections results in a higher stationary level and less difference between vaccinated and naïve mice. Moreover, the addition of different infections in naïve mice increases the global bacillary load and curtails the exponential growth as it reaches earlier the minimum load need to trigger the immune response. Table 1 shows an example of one of the analyses in an attempt to understand this phenomenon. In this case, after only five consecutive infections the global bacillary load in naïve mice stops by day 18, instead of increasing until day 20.

**Table 1:** Example of the analysis considering five MCI with intervals of 24

	CFUs Naïve (non-vaccinated mice)						CFUs BCG-vaccinated mice					
	Infection Sites						Infection Sites					
Day	1	2	3	4	5	Sum	1	2	3	4	5	Sum
**0**	100					100	100					100
**1**	309	100				409	309	100				409
**2**	477	309	100			886	477	309	100			886
**3**	737	477	309	100		1623	737	477	309	100		1623
**4**	1138	737	477	309	100	2762	1138	737	477	309	100	2762
**5**	1759	1138	737	477	309	4420	1759	1138	737	477	309	4420
**6**	2716	1759	1138	737	477	6827	2716	1759	1138	737	477	6827
**7**	4196	2716	1759	1138	737	10546	4196	2716	1759	1138	737	10546
**8**	6481	4196	2716	1759	1138	16290	6481	4196	2716	1759	1138	16290
**9**	10010	6481	4196	2716	1759	25161	10010	6481	4196	2716	1759	25161
**10**	15462	10010	6481	4196	2716	38865	15462	10010	6481	4196	2716	38865
**11**	23883	15462	10010	6481	4196	60032	23883	15462	10010	6481	4196	60032
**12**	36890	23883	15462	10010	6481	92726	36890	23883	15462	10010	6481	92726
**13**	56982	36890	23883	15462	10010	143228	56982	36890	23883	15462	10010	***143228***
**14**	88016	56982	36890	23883	15462	221234	88016	56982	36890	23883	15462	221234
**15**	135952	88016	56982	36890	23883	341724	**135952**	88016	56982	36890	23883	341724
**16**	209995	135952	88016	56982	36890	527835	135952	**135952**	88016	56982	36890	453792
**17**	324364	209995	135952	88016	56982	815309	135952	135952	**135952**	88016	56982	552854
**18**	**501022**	**324364**	**209995**	**135952**	88016	***1259348***	135952	135952	135952	**135952**	88016	631824
**19**	501022	324364	209995	135952	**135952**	1307284	135952	135952	135952	135952	**135952**	679760
**20**	501022	324364	209995	135952	135952	1307284	135952	135952	135952	135952	135952	679760

Evolution of the infection at each site is followed over time using a spreadsheet (all data can be found as Table S1). We consider the formula 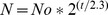 where *No* is the initial dose (100 CFUs), and *t* is the time in days. We highlight in bold italics the total bacillary load in the lung (sum) at the time when the immune response induced the stationary level in the model of single infection (1,195,374 and 135,952 CFUs for naïve and vaccinated mice, respectively). At this time the stationary level is reached at each infection site where there is a minimal bacillary concentration (135,952 CFUs, in bold). This is why, in contrast to what happens in a single infection model, the first infection stops before reaching 1,195,374 CFUs (i.e. on day 18 at 501,022 CFUs). The stationary level is therefore reached at all sites except site 5, which has not reached the minimal bacillary concentration.

In the case of BCG-vaccinated mice, the stationary level at each site could be reached earlier as the sum on day 13 is 143,228. This exceeds the level of 135,952 bacilli, and thus the immune response is ready. However, there is insufficient bacillary load locally, which is why the stationary level is not immediately reached at each infection site. This illustrates how important it is for the local bacillary load to have the benefit of the immune response.

These findings therefore clearly show that, in the context of MCI, a higher number of infections decreases the protection afforded by BCG vaccination.

### Time between infections is critical in MCI

**Figure 3:**
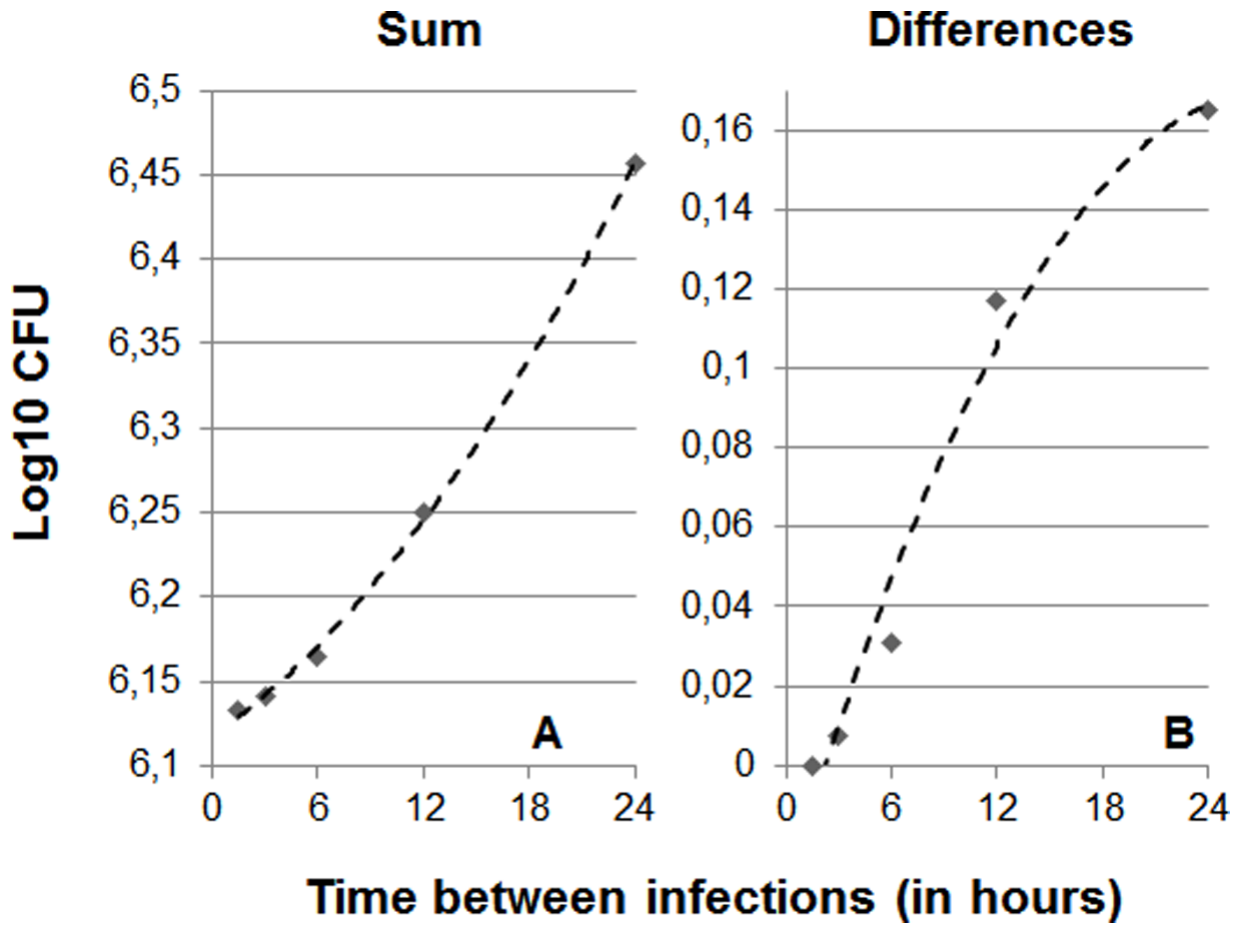
Influence of the interval between multiple consecutive infections (MCI) on the stationary level. Picture A shows the evolution of the total bacillary load in the lung (sum) considering 10 consecutive infections only. The data have been adjusted to a polynomic formula: 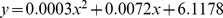 (R^2^ = 0.9988). In Picture B, which show differences between naïve and vaccinated mice, the formula is: 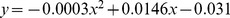 (R^2^ = 0,9774).

The results obtained when simulating infections on a time scale of a few hours show that the lower the interval between infections the lower the maximum overall bacillary load reached. This is due to the fact that, when infections occur at short intervals, the minimal bacillary concentration required to trigger the immune response is reached earlier, thus preventing higher concentrations from being reached as a result of exponential growth (Figure 3). As a result, shorter intervals between infections result in fewer differences between vaccinated and non-vaccinated animals.

## Discussion

Experimental models have taught us that lesions in different evolutionary phases can be observed after a certain period of time [9], [12], [13], a situation that is also found in diseases caused by other pathogens controlled only by cellular immunity [14]. This diversity supports the dynamic hypothesis of latent infection, which is based on a process of constant endogenous reinfection [15] and considers that a certain number of infected macrophages are needed to attract specific lymphocytes, with granulomas being induced as protective structures upon generation of a sufficient local inflammatory response [11].

The mathematical model presented herein is the consequence of the effect of the global bacillary load of the whole lung, as it determines the concentration in the lymph nodes, and thus the ability to trigger an immune response [16], [17]; and the bacillary load at each infection site, which will determine the capacity to attract this immune response and to stop bacillary growth at each site [11].

Our starting point was the experimental data from the mouse model: for a single infection, the bacillary load of a naïve mouse increases until it reaches 1,195,374 bacilli, at which point it stabilizes. Vaccinated mice achieve the stationary level with a lower bacillary load (135,952 bacilli) and much earlier than naive animals [10]. Vaccination with BCG does not prevent Mtb infection in the mouse model, it simply reduces the stationary level of the bacillary load by causing it to be reached earlier. It is important to note that after the first Mtb infection, naïve mice reach a specific immune response as good as that induced by BCG[4]. This is the key to understanding why previous vaccination has less relevance when the animal suffers a number of MCI, or when there is a short interval between them, because naïve mice become immune earlier.

In our experiment, we calculated the overall bacillary loads as well as the bacillary load for every infection simulated, considering each of them to occur at individual sites. Although it is true that the naïve mouse has to mount a proper immune response and vaccinated animals already have such a response (and can therefore act several days earlier), vaccinated animals still need the bacillary load at a specific infection site to exceed a specific level in order to benefit from the effect of the immune response. This is why BCG vaccination cannot avoid MCI. What happens with other TB vaccines? If we extrapolate that the cellular immune response behaves as in the case of BCG, we should expect the same outcomes. However, this may not be the case, which is why it is very important to start to simulate MCI in preclinical experimental modelling to confirm the hypothesis in BCG and, of course, in all the other candidates designed to be better than BCG.

The time intervals in our study were decided arbitrarily in order to mimic the human situation in which a person would be in close contact with a TB patient and therefore submitted to a probable infection, while also taking into account a high incidence and the diagnostic delay. The hours' rate was intended to mimic cohabitation with a TB patient (i.e. household contact), in which contacts are repeated at short time intervals. The daily rate during several days was intended to mimic a constant short contact with a TB patient (i.e. going to have tea served every day by a waiter who suffers from TB).

Although useful, some facts should be taken into account when extrapolating our data to humans. The stationary level for the bacillary load achieved in humans is probably much lower, at around 5 logs [11], [13]. Similarly, the profibrotic environment in human lungs allows encapsulation of the lesions [13] and progressively stops the endogenous reinfection process. This is why, in light of the data obtained in the minipig model (also able to encapsulate the lesions), and in contrast to the situation found in mice, we believe that the stationary level is not that stationary and probably decreases with time, especially after the first two years post-infection [18] although this factor obviously affects both vaccinated and non-vaccinated subjects.

The main conclusion of our study is that MCI reduce the protective effect of BCG, thereby highlighting the need for a better understanding of this factor at both preclinical and clinical levels. MCI is a process that should be taken into account when studying prophylactic interventions, especially when developing candidate vaccines intended to replace BCG. Similarly, greater effort should be invested in promoting research into MCI analysis in different human populations in order to design the best possible prophylactic measures against TB.

## Supporting Information

Table S1 **Data of the evolution of the infection.** Evolution of the infection at each site, followed over time using a spreadsheet (all data included).
(XLS)
